# Identification of the Chinese Population That Can Benefit Most From Postprandial Lipid Testing: Validation of the Use of Oral Fat Tolerance Testing in Clinical Practice

**DOI:** 10.3389/fendo.2022.831435

**Published:** 2022-02-18

**Authors:** Xiaoyu Hou, An Song, Yunpeng Guan, Peipei Tian, Luping Ren, Yong Tang, Chao Wang, Ling Gao, Guangyao Song, Xiaoping Xing

**Affiliations:** ^1^ Department of Internal Medicine, Hebei Medical University, Shijiazhuang, China; ^2^ Department of Endocrinology, Hebei General Hospital, Shijiazhuang, China; ^3^ Key Laboratory of Endocrinology, Ministry of Health, Department of Endocrinology, Peking Union Medical College Hospital, Peking Union Medical College, Chinese Academy of Medical Sciences, Beijing, China; ^4^ Department of Endocrinology, Cangzhou Central Hospital, Cangzhou, China; ^5^ Hebei Key Laboratory of Metabolic Diseases, Hebei General Hospital, Shijiazhuang, China; ^6^ Department of Endocrinology and Metabolism, Shandong Provincial Hospital Affiliated to Shandong University, Jinan, China

**Keywords:** triglyceride, oral fat tolerance test, postprandial lipemia, diabetes, dyslipidemia

## Abstract

**Background:**

Dyslipidemia has become increasingly prevalent in recent decades. Blood lipid concentrations are significantly influenced by diet; however, postprandial triglyceride concentration (PTG) is not often measured. PTG can reflect the risks of diabetes and cardiovascular disease, but not all individuals would benefit from PTG testing.

**Objective:**

The aim of the present study was to determine the PTG response in a Chinese cohort and identify who would benefit from diagnostic PTG measurement.

**Methods:**

A total of 400 Chinese adults were enrolled and underwent oral fat tolerance test (OFTT), which was well tolerated. The participants were assigned to groups according to their fasting triglyceride concentration to evaluate the usefulness of PTG testing. A PTG concentration > 2.5 mmol/L was defined as high (HPTG).

**Results:**

Of the 400 participants, 78.9% showed an undesirable PTG response. Those with FTG ≥1.0 mmol/L had a delayed PTG peak and higher peak values. Seventy-five percent of those with 1.0 mmol/L ≤FTG <1.7 mmol/L had HPTG, of whom 18.6% had impaired glucose tolerance.

**Conclusions:**

The present data confirm the previously reported predictive value of PTG testing. Moreover, the findings indicate that Chinese people with FTGs of 1.0 -1.7 mmol/L may benefit most from the identification of postprandial hyperlipidemia through OFTT because more than half of them have occult HPTG, which may require treatment. Thus, the detection of HPTG using an OFTT represents a useful means of identifying dyslipidemia and abnormal glucose metabolism early.

**Clinical Trial Registration:**

[http://www.chictr.org.cn/index.aspx], identifier ChiCTR1800019514.

## Introduction

Dyslipidemia is a significant risk factor for type 2 diabetes mellitus (T2D) and cardiovascular and cerebrovascular diseases. Owing to lifestyle changes, the incidence of dyslipidemia has markedly increased during recent decades, such that 22.6%–54% of adults in Africa, Southeast Asia, Europe, and the United States, and 45% of Canadian adults have dyslipidemia (1, 2), and according to the World Health Organization (WHO), approximately 2.6 million people die of causes related to dyslipidemia each year. The prevalence of dyslipidemia in China increased rapidly from 18.6% in 2002 to 33.97% in 2016, and the prevalence of hypertriglyceridemia (HTG) was 12.17% (3). The types of dyslipidemia vary among differing ethnicities. The principal manifestations of dyslipidemia in Western countries are high circulating total cholesterol (TC) and low-density lipoprotein-cholesterol (LDL-C) concentrations, whereas in China, dyslipidemia principally manifests as high triglyceride (TG) concentration and low HDL-C concentration (3, 4).

Until 2009, 8–12-h fasting blood samples were recommended for the assessment of lipid profile and cardiovascular risk in most guidelines and statements, mainly because fasting blood lipid concentrations are relatively stable and fairly homogeneous. However, during a normal day, most people are in the fasting state only for a few hours, prior to breakfast. Therefore, it is difficult to comprehensively assess lipid profile by the simple measurement of fasting lipid (FL) concentrations. Furthermore, abnormal postprandial lipid (PL) concentration is an independent risk factor for nutrition-related diseases and obesity (5), and the measurement of PL has many advantages over that of FL, such as the fact that PL can be measured at any time after eating, which reduces the risk of hypoglycemia caused by fasting in patients with diabetes (6).

At present, postprandial triglyceride (PTG) concentration is often used to evaluate PL, mainly because lipid and lipoprotein concentrations are not substantially affected by normal food intake, such that differences in PTG are more indicative than those of other lipids for the assessment of changes in PL (7). PTG is an independent risk factor for cardiovascular diseases and T2D (8–10), and PTG is superior to fasting triglyceride (FTG) concentration for the prediction of cardiovascular risk (11, 12). Since 2009, many countries have issued statements to the effect that PTG represents an alternative to FTG for the prediction of cardiovascular risk (7–10, 13, 14). Previous studies have shown that the early detection and treatment of dyslipidemia can reduce the incidence of cardiovascular disease (11, 12). Therefore, the identification of methods that are suitable for early screening for abnormal lipid metabolism and new therapeutic targets for dyslipidemia should help reducing the incidences of cardiovascular diseases, as well as T2D, and the associated mortality.

To better analyze PL and diagnose dyslipidemia early in people with normal FL, it is necessary to standardize oral fat tolerance test (OFTT). The clinical use of oral glucose tolerance testing (OGTT) has aided the early diagnosis of glucose intolerance and insulin resistance (IR), which has yielded very significant results. Therefore, standardized OFTT may be useful for the evaluation of patients’ lipid metabolism. However, the use of OFTT to evaluate PL is not thought to be essential for every patient. In 2011, a Greek consensus statement recommended that patients with FTGs of 1–2 mmol/L should undergo OFTT (5). Nevertheless, no research data regarding the optimal application of OFTT in China are available.

The first aim of the present study was to identify the FTG range for Chinese patients who would most benefit from the measurement of PL by OFTT. To this end, we evaluated the changes in PL during OFTT and the prevalence of high postprandial triglyceride (HPTG) concentration in patients with a range of FTG concentrations. The second aim was to explore the clinical significance of postprandial OFTT by analyzing the relationship between PTG concentration and glucose tolerance.

## Material and Methods

### Study Sample

This study was approved by the Ethics committee of Hebei General Hospital (approval number: 2018 No.2, date: February 26^th^ 2018) and registered in the Chinese clinical trial registry (registration number: ChiCTR1800019514). Individuals who attended the clinic for routine physical examination volunteered for the study between May 2018 and December 2019. The study complied with the principles of the Declaration of Helsinki. All the volunteers gave their written informed consent and completed a questionnaire that collected basic information, personal history, family history, and the use of medication. The recruited volunteers were between 22 and 70 years old.

### Exclusion Criteria

Vegetarians; patients with chronic diseases, such as heart disease, kidney disease, malignant tumors, or abnormal thyroid function; patients with a family history of endocrine-related diseases, such as familial hypercholesterolemia; patients with acute or chronic hematological diseases or infectious diseases; patients taking drugs that affect glucose or lipid metabolism or inflammation (fish oil, contraceptives, hormones, β-receptor blockers, diuretics, hypoglycemic drugs, and lipid-lowering drugs); and those who had experienced trauma, mental disorders, surgery, stroke, or pregnancy, or a body mass change of >3 kg in the preceding 3 months were excluded from the study.

All the volunteers underwent OGTT, and those with diabetes, according to the criteria for the diagnosis of diabetes published by the Chinese Diabetes Society (15), were excluded (fasting blood glucose (FBG) concentration ≥ 7.0 mmol/L, with or without a 2h-OGTT postprandial blood glucose (PBG) concentration ≥11.1 mmol/L. To avoid extreme values having an excessive influence on the data, patients with FTG > 5.0 mmol/L were excluded.

### Oral Fat Tolerance Test

The choice of total calorie intake was made on the basis that OFTT was designed to last 10 h. On the basis of the OGTT and the advice of a professional nutritionist, the total calorie content of the high-fat meal was 1,500 kcal, of which 60% was provided in the form of fat (monounsaturated fatty acid: polyunsaturated fatty acid: saturated fatty acid ratio = 2:2:1), 20% (300 kcal) as carbohydrate, and 20% (300 kcal) as protein.

All the eligible patients were recommended to continue their normal diet for 1 week before commencing the study, and foods containing high levels of fat or protein were not recommended (All participants received a list of not recommended foods with high fat or high protein the week before the test began). The participants were asked to fast from 22:00 the day before OFTT, which was performed at 08:00. The participants were asked to consume the high-fat meal within 10 minutes, and during the 10-h test, they were permitted to drink only water (All subjects drank freely according to their own conditions), and prohibited from smoking, eating, or performing strenuous exercise. All the participants tolerated the procedure well.

### Laboratory Assays

Blood samples were obtained before, and 2, 4, 6, 8, and 10 h following the high-fat meal. The serum concentrations of TC, TG, and HDL-C were measured using a Hitachi 7600 automatic biochemical analyzer (Hitachi Instruments Ltd., Tokyo, Japan) by a laboratory technician who was blinded to the study details. LDL-C concentration was calculated using the Friedewald formula: LDL-C = TC − (HDL-C) − (TG/5) when the TG concentration was < 4.5 mmol/L (16); otherwise, it was directly measured using the chemical masking method. Fasting insulin (FINS) concentration was measured by electrochemiluminescence. Homeostasis models were used to evaluate IR and ß-cell secretion: homeostasis model assessment-IR (HOMA-IR) = FBG (mmol/L) × FINS (µIU/L)/22.5 (17) and homeostasis model assessment-β-cell function (HOMA-ß) = 20 × FINS (µIU/ml)/[FBG (mmol/ml) − 3.5] (17). The height, body mass, waist circumference (WC), and blood pressure (BP) of all the participants were measured by a single physician.

### Definitions of Clinical Conditions

Consistent with the consensus statement of the 2011 Greek Conference (3), a TG concentration >2.5 mmol/L at any time postprandially was defined as HPTG.

According to the results of OGTT, those with FBG≥6.1 but <7.0 mmol/L, and/or 2h-PBG≥7.8 but <11.1 mmol/L, were defined as prediabetes.

### Grouping of Subjects

Subjects were first allocated to three groups according to their FTG concentration (group A: FTG <1 mmol/L; group B: 1.0 mmol/L ≤ FTG < 2.0 mmol/L; group C: FTG ≥ 2.0 mmol/L) (5).

However, considering that different ethnic groups demonstrate differences in lipid metabolism. In 2006, the Chinese Dyslipidemia Prevention and Treatment society published guidelines for the prevention and treatment of dyslipidemia (18), which recommended that patients with FTGs <1.7 mmol/L should be defined as normal, those with FTGs between 1.7 mmol/L and 2.3 mmol/L should be diagnosed with slightly high FTG, and those with FTG >2.3 mmol/L should be diagnosed as having hypertriglyceridemia. To determine the most appropriate range of FTG for OFTT to be performed in Chinese people, those with FTGs between 1.0 and 2.0 mmol/L were further categorized with reference to the Chinese dyslipidemia standard into the following four groups: Group A: FTG <1.0 mmol/L, Group B-new: 1.0 mmol/L ≤ FTG <1.7 mmol/L, Group C-new: 1.7 mmol/L ≤FTG <2.3 mmol/L, and Group D: FTG >2.3 mmol/L.

### Statistical Analysis

SPSS 21.0 software (IBM Inc., Armonk, NY, USA) was used for the statistical analysis. Numerical data were tested for normality using the Shapiro-Wilk test. Continuous data are expressed as mean ± SD and categorical data are expressed as numbers and percentages. Pearson’s chi-square test was used to compare the prevalence of HPTG and IGT among the groups. Differences in the measured values of the same index at differing time points were analyzed using repeated measures ANOVA. For comparisons among multiple groups, if the data were normally distributed and the variance was homogeneous, one-way ANOVA analysis was used; otherwise, the equivalent non-parametric test was used. If the differences among multiple groups were statistically significant, further pairwise comparisons were performed using Bonferroni. All *P*-values were 2-tailed, and *P* < 0.05 was considered to represent statistical significance.

## Results

### Clinical Characteristics of the Groups With Differing FTG Concentration

A total of 400 patients fulfilled the inclusion and exclusion criteria. On the basis of the 2011 Greek consensus statement (5), the participants were allocated to three groups according to their FTG concentration (group A: FTG <1 mmol/L; group B: 1.0 mmol/L ≤ FTG < 2.0 mmol/L; group C: FTG ≥ 2.0 mmol/L). There were 109 participants in group A (51 men and 58 women, with a mean age of 42 years) 203 in group B (95 men and 108 women, with a mean age of 47 years), and 88 in group C (48 men and 40 women, with a mean age of 48 years).

With increases in FTG concentration, BMI, SBP, DBP, WC, FBG, FINS, HOMA-IR, TC, TG, LDL-C, and apolipoprotein (apo)B increased, and HDL-C decreased (*P* < 0.05). There were no significant differences in total protein (TP), albumin (ALB), globulin (GLB), HOMA-ß, or ApoA1 among the three groups (**Table 1**).

**Table 1:** Basic parameters of groups with different FTG concentration.

Group	A	B	C
	(n=109)	(n=203)	(n=88)
Age (Year)	41.71± 14.75	46.56± 12.48^#^	47.68± 10.38^#^
Sex (Male/Female)	51/58	95/108	48/40
BMI (kg/m^2^)	24.26± 3.54	26.22± 3.59^##^	28.63± 4.13^##**^
SBP (mmHg)	123.13± 14.09	128.04± 16.07^#^	133.3± 13.88^##*^
DBP (mmHg)	75.14± 8.93	78.76± 10.02^#^	83.02± 9.6^##*^
WC (cm)	82.73± 10.83	88.99± 11.26^##^	95.14± 9.99^##**^
TP (g/L)	72.32± 4.52	72.96± 4.51	72.98± 4.55
ALB (g/L)	44.82± 2.58	44.67± 2.32	44.76± 2.49
GLB (g/L)	27.5± 3.68	28.3± 3.91	28.23± 4.07
FBG (mmol/L)	5.26± 0.46	5.66± 1.18^##^	6.17± 1.40^##**^
FINS ((μIU/mL)	9.38± 4.58	11.82± 6.41^#^	15.63± 8.07^##**^
HOMA-IR	2.21± 1.16	3.04± 2.09^##^	4.32± 2.48^##**^
HOMA-ß	112.6± 57.62	124.67± 77.88	135.3± 80.07
TC (mmol/L)	4.24± 0.81	4.79± 0.93^##^	5.30± 1.01^##**^
TG (mmol/L)	0.78± 0.14	1.40± 0.28^##^	2.79± 0.73^##**^
HDL-C(mmol/L)	1.33± 0.29	1.24± 0.26^#^	1.15± 0.25^##*^
LDL-C(mmol/L)	2.60± 0.58	3.06± 0.66^##^	3.43± 0.71^##**^
ApoA1(g/L)	1.46± 0.29	1.43± 0.27	1.39± 0.24
ApoB(g/L)	0.67± 0.15	0.82± 0.19^##^	0.96± 0.20^##**^

^#^P＜0.05,compared with group A. ^##^P＜0.001,compared with group A.^*^P＜0.05,compared with group B. ^**^P＜0.001,compared with group B.

HOMA-IR and HOMA-ß were unitless ratios.

### Changes in Postprandial Circulating Lipid Concentrations in the Three Groups With Differing FTG Concentration

**Figure 1:**
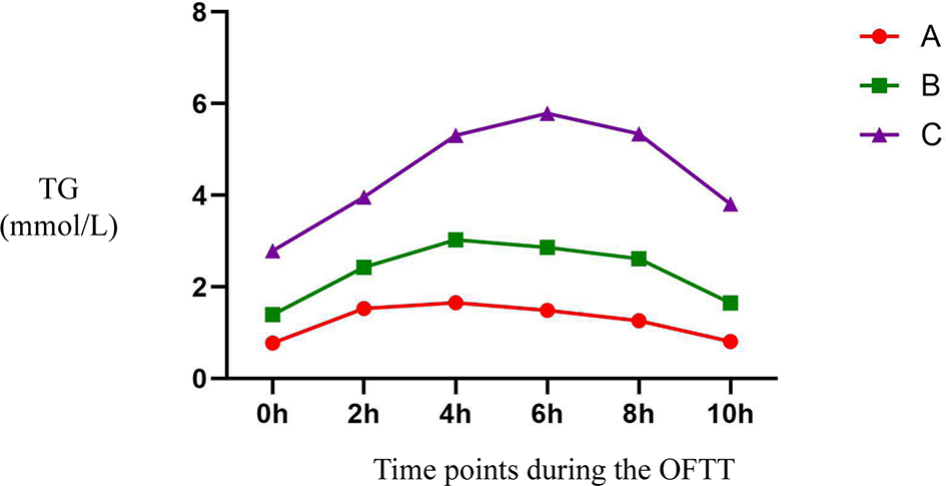
Trend of TG at different time points during the OFTT. Blood was taken at baseline and every 2 hours after the OFTT. The changing trend of triglycerides concentrations were analyzed to determine differences of lipids metabolism among groups with different fasting triglycerides concentrations. All data are expressed as mean.

During OFTT, the TG concentrations of the participants increased gradually and then decreased. The PTGs of groups A and B peaked 4 h postprandially, whereas that of group C peaked 6 h postprandially. Repeated measures ANOVA showed that the TG concentration of group A had returned to its fasting level by 10 h postprandially, whereas those of groups B and C remained higher than the fasting concentrations at this time point (**Figure 1**).

During OFTT, the postprandial TC and LDL-C concentrations of the three groups gradually increased with increases in FTG concentration (*P* < 0.05), whereas that of HDL-C gradually decreased. However, the TC, HDL-C, and LDL-C concentrations were similar in the three groups (**Table 2**).

**Table 2:** Postprandial plasma lipids concentration of groups with different FTG concentration.

	Before	After				
	0h	2h	4h	6h	8h	10h
TC (mmol/L)						
A	4.24± 0.81	4.20± 0.79	4.17± 0.79	4.33± 0.83	4.36± 0.81	4.34± 0.80
B	4.79± 0.93^##^	4.77± 0.91^##^	4.78± 0.91^##^	4.91± 0.94^##^	4.94± 0.95^##^	4.87± 0.91^##^
C	5.30± 1.01^##**^	5.26± 0.98^##**^	5.41± 1.01^##**^	5.58± 1.04^##**^	5.63± 1.02^##**^	5.59± 1.13^##**^
TG (mmol/L)						
A	0.78± 0.14	1.53± 0.44	1.66± 0.61	1.49± 0.62	1.27± 0.50	0.81± 0.29
B	1.40± 0.28^##^	2.43± 0.75^##^	3.03± 1.11^##^	2.86± 1.31^##^	2.62± 1.29^##^	1.65± 0.85^##^
C	2.79± 0.73^##**^	3.97± 1.08^##**^	5.31± 1.42^##**^	5.79± 2.24^##**^	5.35± 2.44^##**^	3.81± 2.11^##**^
HDL-C (mmol/L)						
A	1.33± 0.29	1.32± 0.29	1.27± 0.27	1.29± 0.29	1.33± 0.28	1.32± 0.28
B	1.24± 0.26^#^	1.25± 0.26	1.18± 0.25^#^	1.17± 0.26^#^	1.22± 0.27^#^	1.20± 0.26^#^
C	1.15± 0.25^##*^	1.16± 0.26^##*^	1.10± 0.25^##*^	1.07± 0.26^##*^	1.11± 0.26^##*^	1.10± 0.26^##*^
LDL-C (mmol/L)						
A	2.6± 0.58	2.54± 0.57	2.49± 0.55	2.56± 0.57	2.60± 0.57	2.62± 0.57
B	3.06± 0.66^##^	3.00± 0.66^##^	2.94± 0.63^##^	3.02± 0.64^##^	3.06± 0.66^##^	3.09± 0.66^##^
C	3.43± 0.71^##**^	3.34± 0.66^##**^	3.27± 0.66^##**^	3.33± 0.67^##**^	3.40± 0.67^##**^	3.51± 0.71^##**^

^#^P＜0.05,compared with group A. ^##^P＜0.001,compared with group A.^*^P＜0.05,compared with group B. ^**^P＜0.001,compared with group B.

### Prevalence of HPTG in the Groups With Differing FTG Concentration

**Figure 2:**
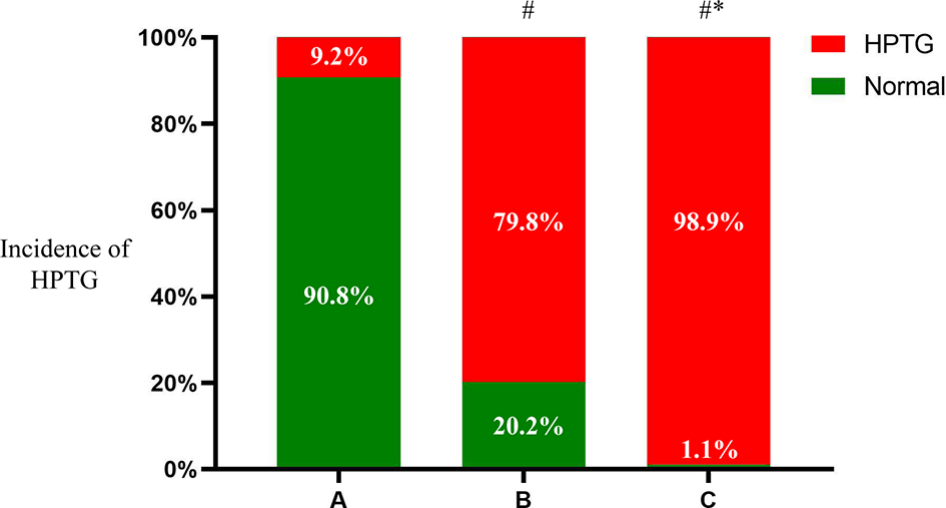
Incidence of HPTG in groups with different FTG concentrations. Incidence of postprandial hypertriglyceridemia were analyzed to evaluate differences among groups with different fasting triglycerides concentrations. All data are expressed as percentage. ^#^P < 0.05 vs. group A. ^*^P < 0.05 vs. group B.

There were significant differences in the prevalence of HPTG among the groups with differing FTG concentration. Ten participants had HPTG in group A (9.2%), 162 had HPTG in group B (79.8%), and 87 had HPTG in group C (98.9%; only one participant had a normal PTG concentration) (**Figure 2**).

### Clinical Parameters of Participants in the Four Groups With Differing FTG Concentration

There were 109 participants in Group A (51 men and 58 women, with a mean age of 42 years), 164 in Group B-new (75 men and 89 women, with a mean age of 46 years) 69 in Group C-new (38 men and 31 women, with a mean age of 49 years), and 58 in Group D (30 men and 28 women, with a mean age of 47 years).

The BMI, SBP, DBP, WC, FBS, FINS, HOMA-IR, TC, TG, LDL-C, and apoB of the four groups increased gradually with FTG concentration, whereas the concentration of HDL-C decreased gradually (all *P* < 0.05). There were no significant differences in TP, ALB, GLB, HOMA-ß, or ApoA1 among the four groups (**Table 3**). The circulating lipid concentrations during OFTT differed among the four groups. Both in the fasting state and at various postprandial time points, the TC and LDL-C concentrations gradually increased with increases in FTG concentration, whereas that of HDL-C gradually decreased. The TC, HDL-C, and LDL-C concentrations showed no significant changes during the OFTT, but the differences among the groups were statistically significant (**Table 4**).

**Table 3:** Basic parameters of groups of different FTG concentration.

Group	A	B-new	C-new	D
	(n=109)	(n=164)	(n=69)	(n=58)
Age (Year)	41.71± 14.75	45.83± 12.90	49.20± 10.52^#^	47.19± 10.03
Sex (Male/Female)	51/58	75/89	38/31	30/28
BMI (kg/m^2^)	24.26± 3.54	25.94± 3.50^#^	27.85± 3.83^##*^	28.71± 4.28^##**^
SBP (mmHg)	123.13± 14.09	126.75± 15.49	131.39± 16.86^#^	135.67± 12.19^##**^
DBP (mmHg)	75.14± 8.93	77.91± 10.10	81.13± 9.67^##^	84.81± 8.68^##**^
WC (cm)	82.73± 10.83	87.71± 10.96^#^	94.42± 10.69^##**^	95.48± 9.85^##**^
TP (g/L)	72.32± 4.52	72.77± 4.55	72.73± 4.41	73.83± 4.52
ALB (g/L)	44.82± 2.58	44.77± 2.34	44.48± 2.58	44.76± 2.20
GLB (g/L)	27.50± 3.68	28.00± 3.87	28.25± 4.01	29.08± 4.07
FBG (mmol/L)	5.26± 0.46	5.65± 1.24^#^	5.86± 1.405^#^	6.22± 1.11^##**&^
FINS ((μIU/mL)	9.38± 4.58	11.54± 6.08^#^	12.81± 6.66^#^	17.21± 8.80^##**&^
HOMA-IR	2.21± 1.16	2.97± 2.10^#^	3.36± 1.93^##^	4.80± 2.67^##**&^
HOMA-ß	112.6± 57.62	124.14± 80.02	126.97± 72.84	139.56± 81.14
TC (mmol/L)	4.24± 0.81	4.78± 0.94^##^	4.96± 0.93^##^	5.38± 1.02^##*^
TG (mmol/L)	0.78± 0.14	1.29± 0.19^##^	1.96± 0.17^##**^	3.13± 0.69^##**&^
HDL-C(mmol/L)	1.33± 0.29	1.26± 0.27	1.15± 0.24^##*^	1.17± 0.25^#^
LDL-C(mmol/L)	2.60± 0.58	3.05± 0.67^##^	3.24± 0.67^##^	3.46± 0.72^##**^
ApoA1(g/L)	1.46± 0.29	1.45± 0.29	1.34± 0.22	1.42± 0.24
ApoB (g/L)	0.67± 0.15	0.81± 0.19^##^	0.88± 0.21^##^	0.97± 0.20^##**^

^#^P < 0.05 vs. group A ^##^P < 0.001 vs. group A. *P < 0.05 vs. group B-new.

**P < 0.001 vs. group B-new & P < 0.05 vs. group C-new. &&P < 0.001 vs. group C-new.

**Table 4:** Plasma lipids concentrations at different time during OFTT of groups with different FTG.

	Before	After				
	0h	2h	4h	6h	8h	10h
TC (mmol/L)						
A	4.24± 0.81	4.20± 0.79	4.17± 0.79	4.33± 0.83	4.36± 0.81	4.34± 0.80
B-new	4.78± 0.94^##^	4.77± 0.92^##^	4.77± 0.92^##^	4.88± 0.96^##^	4.92± 0.98^##^	4.85± 0.92^##^
C-new	4.96± 0.93^##^	4.91± 0.90^##^	5.01± 0.95^##^	5.16± 0.95^##^	5.19± 0.90^##^	5.13± 1.01^##^
D	5.38± 1.02^##**^	5.35± 1.00^##**&^	5.50± 1.03^##**&^	5.69± 1.03^##**&^	5.74± 1.05^##**&^	5.70± 1.12^##**&^
TG (mmol/L)						
A	0.78± 0.14	1.53± 0.44	1.66± 0.61	1.49± 0.62	1.27± 0.50	0.81± 0.29
B-new	1.29± 0.19^##^	2.3± 0.70^##^	2.85± 1.06^##^	2.58± 1.13^##^	2.36± 1.16^##^	1.48± 0.71^##^
C-new	1.96± 0.17^##**^	3.11± 0.72^##**^	4.08± 1.10^##**^	4.32± 1.71^##**^	3.87± 1.48^##**^	2.56± 1.20^##**^
D	3.13± 0.69^##**&&^	4.33± 1.06^##**&&^	5.74± 1.37^##**&&^	6.36± 2.13^##**&&^	5.99± 2.51^##**&&^	4.34± 2.23^##**&&^
HDL-C (mmol/L)						
A	1.33± 0.29	1.32± 0.29	1.27± 0.27	1.29± 0.29	1.33± 0.28	1.32± 0.28
B-new	1.26± 0.27	1.27± 0.27	1.19± 0.26	1.19± 0.26^#^	1.24± 0.28^#^	1.23± 0.26^#^
C-new	1.15± 0.24^##*^	1.15± 0.25^##*^	1.09± 0.25^##*^	1.07± 0.25^##*^	1.12± 0.25^##*^	1.10± 0.23^##*^
D	1.17± 0.25^#^	1.17± 0.25^#^	1.11± 0.24^#^	1.07± 0.25^##*^	1.11± 0.25^##*^	1.10± 0.25^##*^
LDL-C (mmol/L)						
A	2.60± 0.58	2.54± 0.57	2.49± 0.55	2.56± 0.57	2.6± 0.57	2.62± 0.57
B-new	3.05± 0.67^##^	2.99± 0.66^##^	2.93± 0.63^##^	3.00± 0.64^##^	3.05± 0.68^##^	3.08± 0.66^##^
C-new	3.24± 0.67^##^	3.14± 0.64^##^	3.09± 0.63^##^	3.17± 0.63^##^	3.22± 0.62^##^	3.29± 0.68^##^
D	3.46± 0.72^##**^	3.36± 0.68^##*^	3.31± 0.68^##*^	3.35± 0.69^##*^	3.41± 0.68^##*^	3.52± 0.72^##*^

^#^P < 0.05 vs. group A. ^##^P < 0.001 vs. group A. *P < 0.05 vs. group B-new. **P < 0.001 vs. group B-new.

^&^P < 0.05 vs. group C-new. ^&&^P < 0.001 vs. group C-new.

### Trends in TG Concentration During OFTT

**Figure 3:**
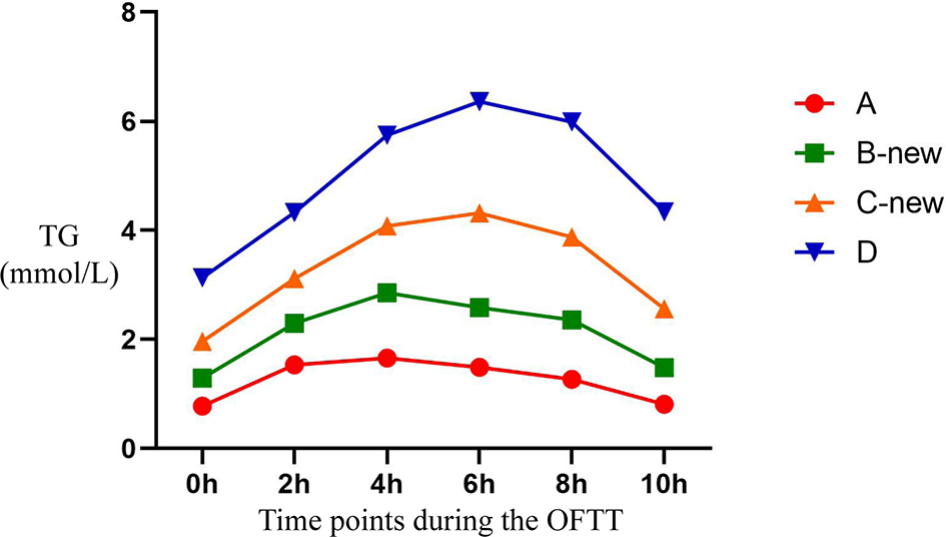
Trend of TG at different time points during the OFTT. Blood was taken at baseline and every 2 hours after the OFTT. The changing trend of triglycerides concentrations were analyzed to determine differences of lipids metabolism among groups with different fasting triglycerides concentrations. All data are expressed as mean.

The TG concentrations in groups A, B-new, C-new, and D increased gradually during OFTT. The concentration peaked 4 h postprandially in group A, but at 6 h in the other groups, and then gradually decreased. There were no significant differences between the PTG and FTG concentrations in groups A and B-new 10 h postprandially, but the PTG concentrations in groups C-new and D were significantly higher than the FTG concentrations 10 h after the meal. There were significant differences in the TG concentrations among the four groups (*P* < 0.001) (**Figure 3**).

### Prevalence of HPTG in the Four Groups

**Figure 4:**
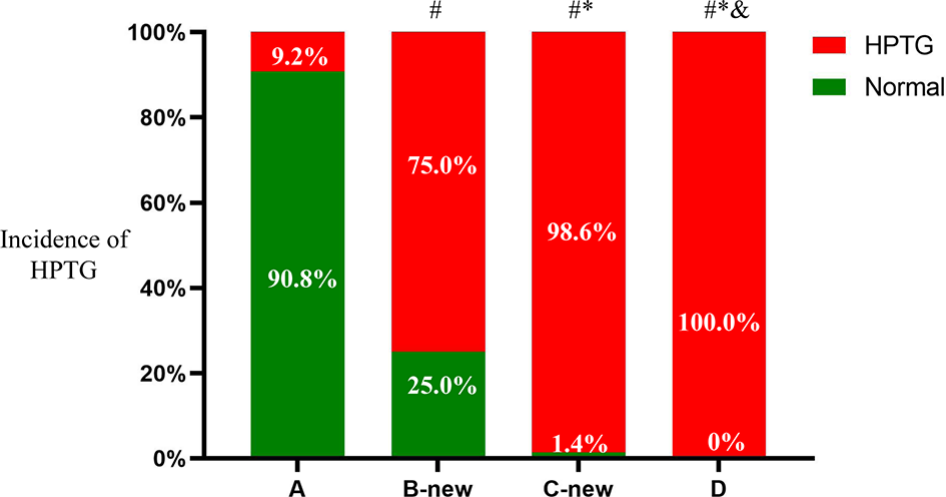
Incidence of HPTG in groups with different FTG concentrations. Incidence of postprandial hypertriglyceridemia were analyzed to evaluate differences among groups with different fasting triglycerides concentrations. All data are expressed as percentage. ^#^P < 0.05 vs. group A. *P < 0.05 vs. group B-new. ^&^P < 0.05 vs. group C-new.

There were 301 participants with HPTG in the four groups, of which 10 were in group A (9.2%), 123 were in group B-new (75.0%), 68 were in group C-new (98.6%), and 100 were in group D (100%). The differences in the prevalence of HPTG among the groups were statistically significant (*P* < 0.05) (**Figure 4**).

To explore the relationship between PTG and the early identification of IGT, the baseline clinical characteristics and prevalence of IGT in the participants with FTG <1.7 mmol/L and FTG <2.0 mmol/L were further analyzed.

### Clinical Characteristics and Prevalence of IGT in Participants With FTG <2.0 mmol/L

On the basis of the consensus recommendations of the Greece conference held in 2011 (5), the participants with FTG <2.0 mmol/L were placed into group F if they had HPTG, whereas those with PTGs in the normal range were placed into group E. There were 332 participants with FTG <2.0 mmol/L, of whom 140 were placed into group E (56 men and 84 women) and 172 were placed into group F (90 men and 82 women). The age, sex, BMI, SBP, DBP, WC, FBG, FINS, HOMA-IR, TC, TG, LDL-C, and apoB of group F were significantly higher than those of group E, whereas the HDL-C and ApoA1 were significantly lower (*P* < 0.05). There were no significant differences in TP, ALB, GLB, or HOMA-ß.

There were 26 patients with IGT in group E (18.6%), whereas the prevalence of IGT in group F was 33.7% (*P* < 0.05) (**Table 5**).

**Table 5:** Basic parameters and incidence of IGT in subjects with different FTG.

Group	E	F	G	H
	(n=140)	(n=172)	(n=140)	(n=133)
Age (Year)	41.68± 14.03	47.46± 12.5^##^	41.68± 14.03	46.82± 13.08^^^
Sex (Male/Female)	56/84	90/82^#^	56/84	70/63^^^
BMI (kg/m^2^)	24.32± 3.43	26.52± 3.60^##^	24.32± 3.43	26.26± 3.52^^^^
SBP (mmHg)	122.66± 13.76	129.31± 16.32^##^	122.66± 13.76	128.09± 15.82^^^
DBP (mmHg)	75.88± 9.01	78.81± 10.23^#^	75.88± 9.01	77.78± 10.37
WC (cm)	83.15± 10.53	89.78± 11.41^##^	83.15± 10.53	88.42± 11.20^^^^
TP (g/L)	72.75± 4.22	72.73± 4.76	72.75± 4.22	72.42± 4.85
ALB (g/L)	44.86± 2.54	44.61± 2.30	44.86± 2.54	44.72± 2.32
GLB (g/L)	27.89± 3.57	28.12± 4.07	27.89± 3.57	27.70± 4.04
FBG (mmol/L)	5.31± 0.53	5.69± 1.25^#^	5.31± 0.53	5.69± 1.33^^^
FINS (μIU/L)	9.70± 5.44	12.00± 6.15^#^	9.70± 5.44	11.70± 5.66^^^
HOMA-IR	2.32± 1.41	3.10± 2.10^##^	2.32± 1.41	3.04± 2.12^^^
HOMA-ß	113.17± 61.53	126.38± 78.53	113.17± 61.53	126.22± 81.33
TC (mmol/L)	4.39± 0.86	4.76± 0.94^##^	4.39± 0.86	4.75± 0.97^^^
TG (mmol/L)	0.89± 0.25	1.41± 0.30^##^	0.89± 0.25	1.29± 0.22^^^^
HDL-C(mmol/L)	1.35± 0.30	1.21± 0.25^##^	1.35± 0.30	1.22± 0.25^^^^
LDL-C(mmol/L)	2.69± 0.60	3.07± 0.68^##^	2.69± 0.60	3.06± 0.69^^^^
ApoA1(g/L)	1.49± 0.31	1.40± 0.24^#^	1.49± 0.31	1.42± 0.25
ApoB(g/L)	0.69± 0.16	0.82± 0.20^##^	0.69± 0.16	0.82± 0.20^^^^
IGT(n%)	26 (18.6%)	58 (33.7%) ^#^	26 (18.6%)	41 (30.8%) ^^^

Group E: Subjects with FTG<2.0 mmol/L and PTG concentrations within range.

Group F: Subjects with FTG<2.0 mmol/L and HPTG concentrations.

Group G: Subjects with FTG<1.7 mmol/L and PTG concentrations within range.

Group H: Subjects with FTG<1.7 mmol/L and HPTG concentrations.

^#^P＜0.05,compared with group E, ^##^P＜0.05,compared with group E.

^^^P＜0.05,compared with group G, ^^^^P＜0.05,compared with group G.

HOMA-IR and HOMA-ß were unitless ratios.

### Clinical Characteristics and Prevalence of IGT in Participants With FTG <1.7 mmol/L

On the basis of the 2016 Chinese guidelines for the prevention and treatment of dyslipidemia (16), participants with FTG <1.7 mmol/L and HPTG were placed into group H and those with PTGs in the normal range were placed into group G. There were 273 participants in groups G and H, of whom 140 were in group G (56 men and 84 women) and 133 were in group H (70 men and 63 women). The age, sex, BMI, SBP, WC, FBG, FINS, HOMA-IR, TC, TG, LDL-C, and apoB of group H were significantly higher than those of group G, whereas HDL-C was significantly lower (all *P* < 0.05). There were no significant differences in DBP, TP, ALB, GLB, HOMA-ß, or apoA1 between the groups.

There were 26 participants with IGT in group G (18.6%) and the prevalence in group H was 30.8% (*P* < 0.05) (**Table 5**).

## Discussion

There is great heterogeneity in serum lipid metabolism among populations. Compared with Caucasians of >20 years old, the concentrations of TC, LDL-C, and HDL-C in Chinese adults are low, whereas that of TG is high. Therefore, we allocated the participants in the present study into groups both according to the FTG classification recommended in the Greek consensus statement (5) and the dyslipidemia standard for Chinese adults (18).

The prevalence of HPTG in the present study was high: of the 400 participants, 301 had HPTG, yielding an overall prevalence of 75.3%. The PTG concentrations of the participants with FTG <1.0 mmol/L were mostly within the normal range, whereas 98.9% of the participants with FTG ≥2.0 mmol/L and all the participants with FTG ≥2.3 mmol/L had HPTG. These findings are consistent with those of previous studies (19, 20). Furthermore, the PTG profiles of participants with FTG ≥1.7 mmol/L had higher and later peaks. On the basis of these results and the Chinese dyslipidemia standard (18), we can conclude that the assessment of PTG using OFTT in Chinese patients with 1.0 ≤FTG <1.7 mmol/L should be of great utility in the early identification of abnormalities in PTG, despite normal fasting blood lipid concentrations.

In the past, blood samples obtained after fasting for 8–12 h were generally used to assess lipid metabolism and evaluate cardiovascular risk for two principal reasons. First, circulating lipid concentrations are substantially affected by diet, and therefore the measurement of FL reduces the effects of differing diet, and is thus more repeatable. However, a study that was conducted over 15 years showed that the PL of individuals changes little (21). Second, the measurement of FL was favored because FTG is required for the calculation of LDL-C using the Friedewald formula (16) when LDL-C concentration cannot be measured directly. However, a study by Langsted et al. showed that LDL-C calculated using the formula is less affected by diet (22).

In recent years, PL has attracted increasing attention and can be used as a supplementary index to FL for more comprehensive assessment of lipid metabolism (7–14). Increases in PTG concentration are principally the result of excess production or poorer postprandial clearance of triglyceride-rich lipoproteins (TRLs), as well as the combined effects of genetic, environmental, and pathological conditions. Such increases in TRLs predispose toward atherosclerosis and cardiovascular disease (CVD) (23, 24).

Many previous studies have shown that PL more accurately reflects the risk of CVD than FL (11, 12, 23, 25, 26) and that PTG is closely associated with metabolic diseases, such as T2D. In healthy Asians, a high PTG concentration has been shown to be an independent risk factor for T2D, independent of FBG (27).

Although many previous studies have shown that PL is a good predictor of CVD and can be used as an alternative to FL, PL have not been widely measured in clinics because of the lack of a standard protocol and widely accepted definition of HPTG. Epidemiological studies performed in Japan showed that the incidences of myocardial infarction, exercise-induced angina pectoris, and sudden cardiac death in people with PTG >1.88 mm/L are significantly higher than in those with lower concentrations (28, 29). A European statement regarding PTG that was published in 2016 suggested that a concentration of 2.0 mmol/L should be used to define HPTG (7). In 2019, researchers at the Second Xiangya Hospital of Central South University in China showed that ≥ 2.0 mmol/L 4 h postprandially is the most appropriate cut-off value of PTG for the diagnosis of HPTG in overweight Chinese people (30). However, in the same year, another study performed in China suggested that a PTG of ≥3.12 mmol/L 4 h postprandially should be used to define HPTG (31).

The consensus statement released by the Greek expert panel in 2011 (5) stated that a PTG >2.5 mmol/L at any time point postprandially should be used to define HPTG. In the present study, we used this to assess the prevalence of HPTG, and further analyzed the clinical parameters of and prevalence of IGT in patients with differing FTG concentration, and found that the prevalence of IGT in participants with HPTG was significantly higher than that in participants in the two groups with normal PTGs. Furthermore, we found that participants with 1.7 ≤ FTG < 2.0 mmol/L had IGT, implying that in people who do not have T2D, despite FTG being normal, a high PTG concentration is also associated with glucose intolerance and a higher risk of T2D. Therefore, the measurement of PTG using OFTT represents a useful means of diagnosing dyslipidemia early and facilitating the early treatment of abnormal glucose and lipid metabolism, to reduce the risk of T2D.

Previous studies have shown that PTG is closely associated with glucose metabolism. Sharrett et al. (32) found that diabetes, obesity, and high FINS and FBG are associated with high FTG and PTG, which is consistent with the high FTG, BMI, FBG, and FINS identified in the present study. PTG was shown to positively correlate with BMI, TC, FBG, and HbA1c in Japanese people (33), and an epidemiological study conducted in China showed that patients with T2DM often have HPTG, even after FBG and FTG have been brought under control (34). Furthermore, the PTG of patients with T2D was found to be significantly higher than that in those with normal glucose tolerance (35), whereas the first-degree relatives of patients with T2D who had normal OGTT profiles had significantly higher PTGs (36), which suggests that dyslipidemia may develop earlier than glucose intolerance.

IR is an important component of the pathogenesis of T2D and atherosclerotic CVD. A study conducted in 2006 showed that high PTG is associated with IR in healthy men (37), and in patients with morbid obesity, the FTG and PTG concentrations increased as IR worsened (38). In the present study, HOMA-IR gradually increased alongside increases in FTG, and the HOMA-IR of the participants with HPTG was significantly higher than that of those with PTGs in the normal range.

The progressive deterioration of islet cell function is another key component of the pathogenesis of T2D. López and coworkers analyzed insulin secretion and IR after a single high-fat meal, and found that the secretion of insulin was high postprandially and that IR was worse (39). In the present study, although there were no significant differences in HOMA-ß among the groups, it gradually increased with increasing FTG. Additionally, the HOMA-ß of participants with HPTG was higher than that of controls. However, the short-term effect of a single high-fat meal on ß-cell function requires further study.

At present, PTG is often used to assess postprandial blood lipid metabolism, mainly because previous studies have shown that the serum TG concentrations during OGTT are substantially affected by diet, whereas other circulating lipid concentrations do not change significantly. PTG increases and then gradually decreases during OFTT (40–42).

In the present study, the PTGs of participants who did not have T2D and had normal FTG concentrations peaked 4 h postprandially, then decreased gradually, returning to the fasting concentrations 10 h postprandially, whereas those of participants with FTG >1.7 mmol/L peaked 6 h postprandially and the concentrations 10 h postprandially were significantly higher than the fasting concentrations, which suggests that even when FL is below the diagnostic threshold for fasting HTG (China > 2.3 mmol/L and elsewhere >2 mmol/L), abnormalities in blood lipids, such as a delayed PTG peak and slow clearance, can already exist.

The timing of the PTG peak in OFTT has varied between studies. Most previous studies have shown that PTG peaks 2–4 h postprandially (31, 43, 44). For example, the 2019 study by Xu et al., conducted at the Second Xiangya Hospital of Central South University in China, showed that OFTT with 800 kcal involved a PTG peak 4 h postprandially (28), and other studies showed PTG peaks 3 h after the meal (40, 44). The timing of the PTG peak during OFTT may be related to the composition of the high-fat diet and the ethnicity of the patients. Standardization of the OFTT protocol requires further research, but in the medium term this may represent a useful means of identifying lipid and glucose metabolic disorders early, thereby facilitating early interventions to reduce the incidence of T2D.

To the best of our knowledge, the present study was the first large study of the use of OFTT to characterize PTG in Chinese people. In this study, we have verified that the FTG range recommended in the Greek consensus statement is appropriate for use in the Chinese population, and further explored the appropriate FTG range in volunteers categorized according to the Chinese dyslipidemia standards. We studied non-diabetic participants, and according to the previous overseas consensus statement, such individuals are able to benefit from the assessment of PTG using OFTT and undergo appropriate interventions (5). However, there were some limitations to the present study, such as all the participants being Chinese adults, and therefore the results may not be applicable to other populations. The amount of drunk water and urination were not registered, which may be a confounding factor. In addition, the timing of postprandial blood collection in the present study was 2 hours after meal ingestion, which may have affected the analysis of the PTG trend. And the evaluation and classification of subjects’ blood glucose metabolism are only based on OGTT and fasting blood glucose, while HbA1C was not considered. This study only included patients without chronic diseases, in the future studies, we’re planning to include patients with chronic diseases and who take lipid lowering drugs.

In conclusion, we have shown that OFTT is useful for the identification of dyslipidemia in individuals with FTGs of 1.0–2.0 mmol/L. Furthermore, according to the Chinese dyslipidemia standard, people with FTGs of 1.0–1.7 mmol/L may benefit most from OFTT.

## Data Availability Statement

The raw data supporting the conclusions of this article will be made available on reasonable request. Please contact the corresponding author.

## Ethics Statement

The studies involving human participants were reviewed and approved by Ethics committee of Hebei General Hospital. The patients/participants provided their written informed consent to participate in this study.

## Author Contributions

XH: Formal analysis, Writing-Original draft, Data processing. AS: Acquisition of data, Project administration, Data processing. YG: Acquisition of data, Project administration, Methodology. PT: Project administration, Methodology. LR, YT, CW, and LG: Conceptualization, Supervision, Methodology, Supervision, Writing-Reviewing. GS and XX: Conceptualization, Methodology, Supervision, Writing-Reviewing, and Editing. All authors reviewed the manuscript and approved the final version.

## Funding

This work was supported by the Research Fund of Hebei Provincial Government.

## Author Disclaimer

The content is solely the responsibility of the authors and does not necessarily represent the official views of the Hebei Provincial Government.

## Conflict of Interest

The authors declare that the research was conducted in the absence of any commercial or financial relationships that could be construed as a potential conflict of interest.

## Publisher’s Note

All claims expressed in this article are solely those of the authors and do not necessarily represent those of their affiliated organizations, or those of the publisher, the editors and the reviewers. Any product that may be evaluated in this article, or claim that may be made by its manufacturer, is not guaranteed or endorsed by the publisher.

## Abbreviations

WHO, World Health Organization; HTG, hypertriglyceridemia; TC, total cholesterol; TG, triglycerides; FL, fasting lipid; PL, postprandial lipid; PTG, postprandial triglyceride; FTG, fasting triglyceride; OFTT, oral fat tolerance test; OGTT, oral glucose tolerance testing; IR, insulin resistance; HPTG, high postprandial triglyceride; FBG, fasting blood glucose; PBG, postprandial blood glucose; FINS, fasting insulin; HOMA, homeostasis model assessment; HOMA-β, homeostasis model assessment-β-cell function; WC, waist circumference; BP, blood pressure; IGT, impaired glucose tolerance; Apo, apolipoprotein; TP, total protein; ALB, albumin; GLB, globulin; TRLs, triglyceride-rich lipoproteins; NS, not significant.

## References

[1] Nikparvar M Khaladeh M Yousefi H Vahidi Farashah M Moayedi B Kheirandish M. Dyslipidemia and Its Associated Factors in Southern Iranian Women, Bandare-Kong Cohort Study, A Cross-Sectional Survey. Sci Rep (2021) 11(1):9125. doi: 10.1038/s41598-021-88680-z 33911149 PMC8080669

[2] Joffres M Shields M Tremblay MS Connor Gorber S. Dyslipidemia Prevalence, Treatment, Control, and Awareness in the Canadian Health Measures Survey. Can J Public Health (2013) 104(3):e252–7. doi: 10.17269/cjph.104.3783 PMC6974261 23823891

[3] Pan L Yang Z Wu Y Yin RX Liao Y Wang J. The Prevalence, Awareness, Treatment and Control of Dyslipidemia Among Adults in China. Atherosclerosis (2016) 248:2–9. doi: 10.1016/j.atherosclerosis.2016.02.006 26978581

[4] Li JH Wang LM Li YC Bi YF Jiang Y Mi SQ. Epidemiologic Characteristics of Dyslipidemia in Chinese Adults 2010. Zhonghua Yu Fang Yi Xue Za Zhi (2012) 46(5):414–8. doi: 10.3760/cma.j.issn.0253-9624.2012.05.008 22883727

[5] Kolovou GD Mikhailidis DP Kovar J Lairon D Nordestgaard BG Ooi TC. Assessment and Clinical Relevance of Non-Fasting and Postprandial Triglycerides: An Expert Panel Statement. Curr Vasc Pharmacol (2011) 9(3):258–70. doi: 10.2174/157016111795495549 21314632

[6] Langsted A Nordestgaard BG. Nonfasting Versus Fasting Lipid Profile for Cardiovascular Risk Prediction. Pathology (2019) 51(2):131–41. doi: 10.1016/j.pathol.2018.09.062 30522787

[7] Nordestgaard BG Langsted A Mora S Kolovou G Baum H Bruckert E. Fasting Is Not Routinely Required for Determination of a Lipid Profile: Clinical and Laboratory Implications Including Flagging at Desirable Concentration Cut-Points–A Joint Consensus Statement From the European Atherosclerosis Society and European Federation of Clinical Chemistry and Laboratory Medicine. Eur Heart J (2016) 37:1944–58. doi: 10.1093/eurheartj/ehw152 PMC4929379 27122601

[8] Nordestgaard BG Hilsted L Stender S. Plasma Lipids in Non-Fasting Patients and Signal Values of Laboratory Results. (Danish) Ugeskr Laeger (2009) 171:1093. 19321088

[9] Miller M Stone NJ Ballantyne C Bittner V Criqui MH Ginsberg HN. Triglycerides and Cardiovascular Disease: A Scientific Statement From the American Heart Association. Circulation (2011) 123:2292–333. doi: 10.1161/CIR.0b013e3182160726 21502576

[10] National Institute for Health and Care Excellence (NICE). Cardiovascular Disease: Risk Assessment and Reduction, Including Lipid Modification. In: Clinical Guideline [CG181]. London: NICE (2014). 36952507

[11] Bansal S Buring JE Rifai N Mora S Sacks FM Ridker PM. Fasting Compared With Nonfasting Triglycerides and Risk of Cardiovascular Events in Women. . JAMA (2007) 298(3):309–16. doi: 10.1001/jama.298.3.309 17635891

[12] Lindman AS Veierød MB Tverdal A Pedersen JI Selmer R. Nonfasting Triglycerides and Risk of Cardiovascular Death in Men and Women From the Norwegian Counties Study. Eur J Epidemiol (2010) 25(11):789–98. doi: 10.1007/s10654-010-9501-1 PMC2991549 20890636

[13] Downs JR O’Malley PG. Management of Dyslipidemia for Cardiovascular Disease Risk Reduction: Synopsis of the 2014 US Department of Veterans Affairs and US Department of Defense Clinical Practice Guideline. Ann Intern Med (2015) 163:291–7. doi: 10.7326/M15-0840 26099117

[14] Catapano AL Graham I De Backer G Wiklund O Chapman MJ Drexel H. ESC/EAS Guidelines for the Management of Dyslipidemias. Eur Heart J (2016) 37:2999–3058. doi: 10.1093/eurheartj/ehw272 27567407

[15] Chinese Diabetes Society. Chinese Guideline for the Prevention and Treatment of Type 2 Diabetes Mellitus (2017 Edition). Chin J Diabetes Mellitus (2018) 10(1):4–67. doi: 10.3760/cma.j.issn.1674-5809.2018.01.003

[16] Lee J Jang S Jeong H Ryu OH. Validation of the Friedewald Formula for Estimating Low Density Lipoprotein Cholesterol: The Korea National Health and Nutrition Examination Survey, 2009 to 2011. Korean J Intern Med (2020) 35(1):150–9. doi: 10.3904/kjim.2017.233 PMC6960042 29551052

[17] Matthews DR Hosker JP Rudenski AS Naylor BA Treacher DF Turner RC. Homeostasis Model Assessment: Insulin Resistance and Beta-Cell Function From Fasting Plasma Glucose and Insulin Concentrations in Man. Diabetologia (1985) 28(7):412–9. doi: 10.1007/BF00280883 3899825

[18] Joint Committee Issued Chinese Guideline for the Management of Dyslipidemia in a: [2016 Chinese Guideline for the Management of Dyslipidemia in Adults]. Zhonghua Xin Xue Guan Bing Za Zhi (2016) 44:833–53. doi: 10.3969/j.issn.1000-3614.2016.10.001 27903370

[19] Mihas C Kolovou GD Mikhailidis DP Kovar J Lairon D Nordestgaard BG. Diagnostic Value of Postprandial Triglyceride Testing in Healthy Subjects: A Meta-Analysis. Curr Vasc Pharmacol (2011) 9(3):271–80. doi: 10.2174/157016111795495530 21314631

[20] Perez-Martinez P Alcala-Diaz JF Kabagambe EK Garcia-Rios A Tsai MY Delgado-Lista J. Assessment of Postprandial Triglycerides in Clinical Practice: Validation in a General Population and Coronary Heart Disease Patients. J Clin Lipidol (2016) 10(5):1163–71. doi: 10.1016/j.jacl.2016.05.009 PMC7025632 27678433

[21] Freiberg JJ Tybjaerg-Hansen A Jensen JS Nordestgaard BG. Nonfasting Triglycerides and Risk of Ischemic Stroke in the General Population. JAMA (2008) 300(18):2142–52. doi: 10.1001/jama.2008.621 19001625

[22] Langsted A Nordestgaard BG. Nonfasting Lipids, Lipoproteins, and Apolipoproteins in Individuals With and Without Diabetes: 58 434 Individuals From the Copenhagen General Population Study. Clin Chem (2011) 57(3):482–9. doi: 10.1373/clinchem.2010.157164 21189274

[23] Nordestgaard BG Benn M Schnohr P Tybjaerg-Hansen A. Nonfasting Triglycerides and Risk of Myocardial Infarction, Ischemic Heart Disease, and Death in Men and Women. JAMA (2007) 298(3):299–308. doi: 10.1001/jama.298.3.299 17635890

[24] Farukhi ZM Demler OV Caulfield MP Kulkarni K Wohlgemuth J Cobble M. Comparison of Nonfasting and Fasting Lipoprotein Subfractions and Size in 15,397 Apparently Healthy Individuals: An Analysis From the VITamin D and OmegA-3 Tria. J Clin Lipidol (2020) 14(2):241–51. doi: 10.1016/j.jacl.2020.02.005 PMC7166180 32205068

[25] Mora S Rifai N Buring JE Ridker M. Fasting Compared With Nonfasting Lipids and Apolipoproteins for Predicting Incident Cardiovascular Events . Circulation (2008) 118(10):993–1001. doi: 10.1161/CIRCULATIONAHA.108.777334 18711012 PMC2574817

[26] Kajikawa M Maruhashi T Matsumoto T Iwamoto Y Iwamoto Y Oda N. Relationship Between Serum Triglyceride Levels and Endothelial Function in a Large Community-Based Stud . Atherosclerosis (2016) 249:70–5. doi: 10.1016/j.atherosclerosis.2016.03.035 27065244

[27] Nishikawa T Okamura T Shima A Kawatsu Y Sugiyama D Kadota A. Casual Serum Triglyceride as a Predictor of Premature Type 2 Diabetes Mellitus: An 8-Year Cohort Study of Middle-Aged Japanese Workers. Diabetol Int (2016) 7(3):252–8. doi: 10.1007/s13340-015-0241-z PMC6224993 30603271

[28] Iso H Naito Y Sato S Kitamura A Okamura T Sankai T. Serum Triglycerides and Risk of Coronary Heart Disease Among Japanese Men and Women. Am J Epidemiol (2001) 153(5):490–9. doi: 10.1093/aje/153.5.490 11226981

[29] Teramoto T Sasaki J Ishibashi S Birou S Daida H Dohi S. Executive Summary of the Japan Atherosclerosis Society (JAS) Guidelines for the Diagnosis and Prevention of Atherosclerotic Cardiovascular Diseases in Japan -2012 Version. J Atheroscler Thromb (2013) 20(6):517–23. doi: 10.5551/jat.15792 23665881

[30] Tian F Xiang QY Zhang MY Chen YQ Lin QZ Wen T. Changes in non-Fasting Concentrations of Blood Lipids After a Daily Chinese Breakfast in Overweight Subjects Without Fasting Hypertriglyceridemia. Clin Chim Acta (2019) 490:147–53. doi: 10.1016/j.cca.2019.01.004 30615853

[31] Xu J Chen YQ Zhao SP Liu L. Determination of Optimal Cut-Off Points After a High-Fat Meal Corresponding to Fasting Elevations of Triglyceride and Remnant Cholesterol in Chinese Subjects. Lipids Health Dis (2019) 18(1):206. doi: 10.1186/s12944-019-1146-9 31767005 PMC6876091

[32] Sharrett AR Heiss G Chambless LE Boerwinkle E Coady SA Folsom AR. Metabolic and Lifestyle Determinants of Postprandial Lipemia Differ From Those of Fasting Triglycerides: The Atherosclerosis Risk In Communities (ARIC) Study. Arterioscler Thromb Vasc Biol (2001) 21(2):275–81. doi: 10.1161/01.ATV.21.2.275 11156865

[33] Kobayashi J Nohara A Mabuchi H Kawashiri MA Miyazaki O Inazu A. The Distribution of Fasting and Non-Fasting Serum Triglyceride Levels in Japanese Population. Clin Chim Acta (2006) 374(1-2):173–5. doi: 10.1016/j.cca.2006.06.032 16890926

[34] Lipid metabolism group of Endocrinology branch of Chinese Medical Association. Expert Consensus on Prevention and Treatment of Type 2 Diabetes Mellitus Combined With Dyslipidemia in China (Revised Edition 2017). Chin J Endocrinol Metab (2017) 33(11):925–36. doi: 10.3760/cma.j.issn.1000-6699.2017.11.004

[35] Madhu S Sinha B Aslam M Mehrotra G Dwivedi S. Postprandial Triglyceride Responses and Endothelial Function in Prediabetic First-Degree Relatives of Patients With Diabetes. J Clin Lipidol (2017) 11(6):1415–20. doi: 10.1016/j.jacl.2017.08.001 28867451

[36] Deng L Liu S Gong Y Tian H Tian H Song J. Increased Metabolic Disorders and Impaired Insulin Secretory Function in the First-Degree Relatives of Type 2 Diabetic Patients With Normal Glucose Tolerance. Metab Syndr Relat Disord (2016) 14(9):431–6. doi: 10.1089/met.2016.0002 27689409

[37] Pedrini MT Niederwanger A Kranebitter M Tautermann C Ciardi C Tatarczyk T. Postprandial Lipaemia Induces an Acute Decrease of Insulin Sensitivity in Healthy Men Independently of Plasma NEFA Levels. Diabetologia (2006) 49(7):1612–8. doi: 10.1007/s00125-006-0262-z 16752179

[38] Tinahones FJ Murri-Pierri M Garrido-Sánchez L Tautermann C Ciardi C Tatarczyk T. Oxidative Stress in Severely Obese Persons is Greater in Those With Insulin Resistance. Obes (Silver Spring) (2009) 17(2):240–6. doi: 10.1038/oby.2008.536 19023278

[39] López S Bermúdez B Pacheco YM Villar J Abia R Muriana FJ. Distinctive Postprandial Modulation of Beta Cell Function and Insulin Sensitivity by Dietary Fats: Monounsaturated Compared With Saturated Fatty Acids. Am J Clin Nutr (2008) 88(3):638–44. doi: 10.1093/ajcn/88.3.638 18779278

[40] Masuda D Nakagawa-Toyama Y Nakatani K Inagaki M Tsubakio-Yamamoto K Tsubakio-Yamamoto K. Ezetimibe Improves Postprandial Hyperlipidaemia in Patients With Type IIb Hyperlipidaemia. Eur J Clin Invest (2009) 39(8):689–98. doi: 10.1111/j.1365-2362.2009.02163.x 19490064

[41] Yunoki K Nakamura K Miyoshi T Enko K Kohno K Morita H. Ezetimibe Improves Postprandial Hyperlipemia and its Induced Endothelial Dysfunction. Atherosclerosis (2011) 217(2):486–91. doi: 10.1016/j.atherosclerosis.2011.04.019 21592480

[42] Ai M Tanaka A Ogita K Sekinc M Numano F Numano F. Relationship Between Plasma Insulin Concentration and Plasma Remnant Lipoprotein Response to an Oral Fat Load in Patients With Type 2 Diabetes. J Am Coll Cardiol (2001) 38(6):1628–32. doi: 10.1016/S0735-1097(01)01611-4 11704373

[43] Sevilla-González MDR Aguilar-Salinas CA Muñóz-Hernández L Almeda-Valdés P Mehta R Zubirán R. Identification of a Threshold to Discriminate Fasting Hypertriglyceridemia With Postprandial Values. Lipids Health Dis (2018) 17(1):156. doi: 10.1186/s12944-018-0803-8 30021651 PMC6052549

[44] Leon-Acuña A Torres-Peña JD Alcala-Diaz JF Almeda-Valdés P Mehta R Zubirán R. Lifestyle Factors Modulate Postprandial Hypertriglyceridemia: From the CORDIOPREV Study. Atherosclerosis (2019) 290:118–24. doi: 10.1016/j.atherosclerosis.2019.09.025 31605877

